# Supplementary material for: A generalized architecture of quantum secure direct communication for N disjointed users with authentication
Source: Sci Rep. 2015 Nov 18;5:16080. doi: 10.1038/srep16080 (PMC4649760; doi:10.1038/srep16080)
Supplement: Supplementary Information [file srep16080-s1.pdf]

A generalized architecture of quantum secure direct communication for N disjointed users with authentication

Ahmed Farouk, Magdy Zakria, Adel Megahed and Fatma Omara

Supplementary Information

1 Communication Process between Two Disjoint Users with Partial and Full Cooperation of Quantum Server

1.1 Partial Cooperation Process

Here we will use the property of dense coding for encoding and transmitting a serial of classical messages between two disjoint users with partial support of the quantum server. In other words according to quantum server's publication and received user's measurement. In this scenario u_i informs the quantum server about his request to transmit a message to distant u_j . The quantum server creates a *GHZ* three particle states $|\Psi_{iqj}>$ where *i* represents particle state for the transmitted user u_i , j represents particle state for the received user u_j and q represents particle state for quantum server. u_i prepares his plain message which consists of classical bits. According to the value of transmitted message classical bits, u_i performs one of the unitary transformations on his qubit of the entangled *GHZ* three particle states $|\Psi_{iqj}>$. After that the *GHZ* states will be converted according to transmitted bits and u_i transformation. Thenceforth, u_i transmits the transformed message to the received disjoint user $u_j. u_j$ retrieves the original sent secret message by applying his bell measurements' and the quantum server's publication on the received encoded message. Fig. S1 illustrates the flow processes to communicate disjoint user 1 and user 2 with partial support of the quantum server. The required steps listed below with supportive equations see (Eq. $(1-3)$).

1. The quantum server creates a *GHZ* three particle states secret key sequence $|\Psi_i>$

$$|\Psi_i> = \{ \Psi_1, \Psi_2, \Psi_3, \ldots\ldots\ldots\ldots\ldots\ldots\ldots\ldots\ldots\ldots.., \Psi_N \} \quad (1)$$

Suppose that

$$\Psi_i = \frac{1}{\sqrt{2}} (|000_{iqj}> + |111_{iqj}>) \text{ where } 1 \le i \le N \quad (2)$$

$$\Psi_{iqj} = \frac{1}{\sqrt{2}} \left(|\Phi^+>_{ij} |+>_q + |\Phi^->_{ij} |->_q \right) ,$$

$$\text{As } |+> = \frac{1}{\sqrt{2}} (|0> + |1>), |-> = \frac{1}{\sqrt{2}} (|0> - |1>) \quad (3)$$

2. The disjoint user u_i chooses a randomly subset of GHZ particle sequences Ψ and keeps it confident
3. u_i generates a random sequence bits string of transmitted plain message. According to each two transmitted bits which $(00, 01, 10, 11)$, The disjoint user u_i applying one of the unitary transformation operations $\breve{U} = \{ \breve{U}_1, \breve{U}_2, \breve{U}_3, \breve{U}_4 \}$ which corresponds to four Pauli operations $\{ I, X, Y, Z \}$ respectively.
4. Afterward, the *GHZ* states will be converted according to transmitted bits and u_i transformation as illustrated by (Eq. $(4)-(7)$).

   - When the transmitted two bits = 00, u_i applies *I* operation on his bit

$$I_{u_i}|\Psi> = \frac{1}{\sqrt{2}}\left(|000>_{iqj} + |111>_{iqj}\right)$$

$$= ½\left\{\left(|\Phi^+>_{ij} + |\Phi^->_{ij}\right)|0>_q + \left(|\Phi^+>_{ij} - |\Phi^->_{ij}\right)|1>_q\right\}$$

$$= \frac{1}{\sqrt{2}}\left(|\Phi^+>_{ij}|+>_q + |\Phi^->_{ij}|->_q\right) \tag{4}$$

- When the transmitted two bits = 01, u_i applies X operation on his bit

$$X_{u_i}|\Psi> = \frac{1}{\sqrt{2}}\left(|100>_{iqj} + |011>_{iqj}\right)$$

$$= ½\left\{\left(|\psi^+>_{ij} - |\psi^->_{ij}\right)|0>_q + \left(|\psi^+>_{ij} + |\psi^->_{ij}\right)|1>_q\right\}$$

$$= \frac{1}{\sqrt{2}}\left(|\psi^+>_{ij}|+>_q - |\psi^->_{ij}|->_q\right) \tag{5}$$

- When the transmitted two bits = 10, u_i applies Y operation on his bit

$$Y_{u_i}|\Psi> = \frac{1}{\sqrt{2}}\left(|011>_{iqj} - |100>_{iqj}\right)$$

$$= ½\left\{\left(|\psi^+>_{ij} + |\psi^->_{ij}\right)|1>_q - \left(|\psi^+>_{ij} - |\psi^->_{ij}\right)|0>_q\right\}$$

$$= \frac{1}{\sqrt{2}}\left(|\psi^->_{ij}|+>_q - |\psi^+>_{ij}|->_q\right) \tag{6}$$

- When the transmitted two bits = 11, u_i applies Z operation on his bit

$$Z_{u_i}|\Psi> = \frac{1}{\sqrt{2}}\left(|000>_{iqj} - |111>_{iqj}\right)$$

$$= ½\left\{\left(|\Phi^+>_{ij} + |\Phi^->_{ij}\right)|0>_q - \left(|\Phi^+>_{ij} - |\Phi^->_{ij}\right)|1>_q\right\}$$

$$= \frac{1}{\sqrt{2}}\left(|\Phi^->_{ij}|+>_q + |\Phi^+>_{ij}|->_q\right) \tag{7}$$

5. After applying the proper *GHZ* transformation, u_i transmits the encoded message to the received disjoint user u_j
6. u_j performs a Bell measurement on his particle and u_i particle , as well , the quantum server calculates the status of his particle according to x basis $\{+,-\}$ and announces his measurement results.
7. u_j uses his measurements' and the quantum server's publication for retrieving the original sent secret bits by u_i

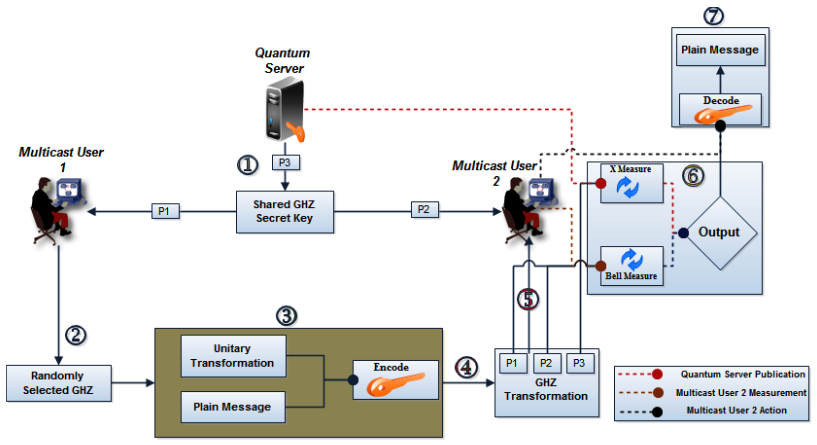

Figure S1: Communication Process between Two Disjoint Users with Partial Support of Quantum Server "drawn by A.F"

1.2 Full Cooperation Process

This process consists of the steps required for transmission of classical message between two disjoint users with full support of the quantum server. In other words the quantum server is functioning as message passing center between the communicated disjoint users. To accomplish this function, u_i transmits the transformed message to the quantum server instead of u_j. Afterwards, u_j retrieves the original sent secret message according to his publication and quantum server's bell measurement on the received transformed message. Fig. S2 illustrates the flow processes to communicate disjoint user 1 and user 2 with full support of the quantum server. The required steps listed below as the first three steps are same partial mechanism but the process is just changed from step five as indicated in Fig. S2 green box.

5. After applying the proper GHZ transformation, u_i transmits the encoded message to the quantum server.
6. The quantum server performs a Bell measurement his particle and u_i particle. u_j calculates the status of his particle according to x basis $\{+\ ,-\}$ and announces his measurement results.
7. u_j employs quantum server's measurement and his publication for retrieving the original sent secret bits by u_i as shown in Table S1. For example when u_j publication is equivalent to $|+>$ and Quantum Server's measurement is $|\Phi^- >$, so u_j can conclude that u_i applied Z operation and the sent bits are 11.

Table S1: Correlation between Quantum Server's Measurement, u_j Publication, u_i Operation and Sent Bits

Quantum Server's Measurement	$\boldsymbol{u_j}$ *Publication*	$\boldsymbol{u_i}$ *Operation*	*Sent Bits*
$\|\boldsymbol{\Phi}^+ >_{iq}$	$\|+>_j$	I	00
$\|\boldsymbol{\Phi}^+ >_{iq}$	$\|->_j$	Z	11
$\|\boldsymbol{\psi}^+ >_{iq}$	$\|+>_j$	X	01
$\|\boldsymbol{\psi}^+ >_{iq}$	$\|->_j$	Y	10
$\|\boldsymbol{\Phi}^- >_{iq}$	$\|+>_j$	Z	11
$\|\boldsymbol{\Phi}^- >_{iq}$	$\|->_j$	I	00
$\|\boldsymbol{\psi}^- >_{iq}$	$\|+>_j$	X	10
$\|\boldsymbol{\psi}^- >_{iq}$	$\|->_j$	Y	01

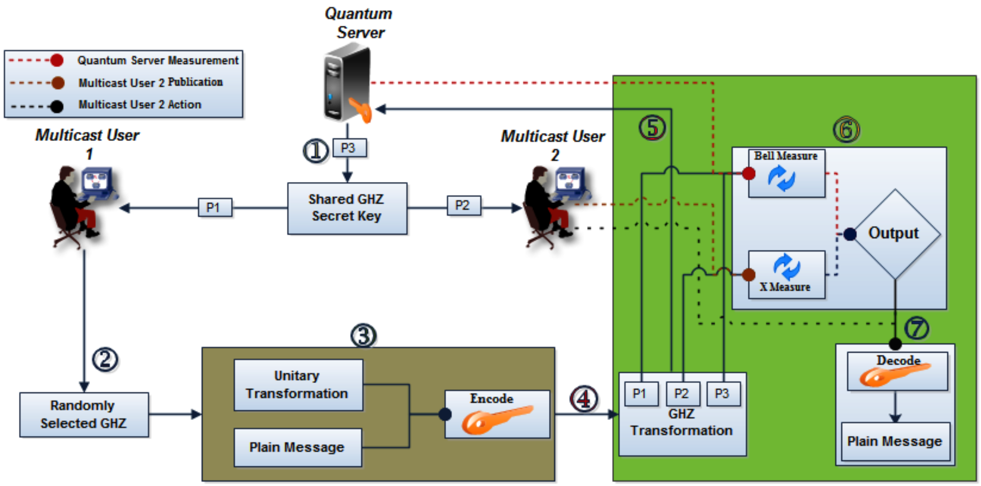

Figure S2: Communication Process between Two Disjoint Users with Full Support of Quantum Server: Green Box indicates Different Steps from Fig. S1 "drawn by A.F"

2 Communication Process between Three Disjoint Users with Partial and Full Cooperation of Quantum Server

2.1 Partial Cooperation Process

This process consists of the steps required when two disjointed disjoint users u_i and u_j would like to transmit a secret message to a remote user u_l with partial support of the quantum server. In this scenario the quantum server creates a three particles of four particles GHZ state $|\Psi_{ijql}>$ and transmits it to u_i , u_j and u_l . Both u_i and u_j prepares their plain message which consists of classical bits. According to the value of transmitted message classical bits, both u_i and u_j applies one of their specified unitary transformation, each on his corresponding qubit of the entangled GHZ four particle states $|\Psi_{ijql}>$ as shown in Table (S2 , S3). After that the GHZ states will be converted according to transmitted bits, u_i and u_j transformations. Thenceforth, u_i and u_j sent the transformed message to a remote user u_l . Afterwards, u_l retrieves the original sent secret message by applying his GHZ measurements' and the quantum server's publication on the received transformed message. Fig. S3 illustrates the flow processes to communicate disjoint user 1, user 2 and user 3 with partial support of the quantum server. The required steps listed below with supportive equations from Eq. (8−10).

1. The quantum server creates a four particles GHZ state secret key $|\Psi_i>$

$$|\Psi_i> = \{ \Psi_1, \Psi_2 , \Psi_3 , \dots \dots \dots \dots \dots \dots \dots \dots \dots .., \Psi_N \} \quad (8)$$

Suppose that

$$\Psi_i = \frac{1}{\sqrt{2}} (|0000_{ijql}> + |1111_{ijql}>) \text{ where } 1 \leq i \leq N \quad (9)$$

$$\Psi_i = \frac{1}{\sqrt{2}} \left(|\Psi^+ >_{ijl} |+>_q + |\Psi^- >_{ijl} |->_q \right) ,$$

$$\text{As } |+> = \frac{1}{\sqrt{2}} (|0> + |1>) , |-> = \frac{1}{\sqrt{2}} (|0> - |1>) \quad (10)$$

2. Both u_i and u_j choose a randomly subset of GHZ particle sequences Ψ and keep it confident
3. u_i generates a random sequences bits string of transmitted plain message. According to each two transmitted bits which $(00, 01, 10, 11)$, The disjoint user u_i applying one of the unitary transformation operations $\breve{U} = \{ \breve{U}_1 , \breve{U}_2 , \breve{U}_3 , \breve{U}_4 \}$ which corresponds to four Pauli operations $\{ I , X , Y , Z \}$ respectively as shown in Table S2. u_j generates a random sequences bits string of transmitted plain message. u_j applying $I_{u_j}|\Psi>$ or $X_{u_j}|\Psi>$ according to the value of particle 0 or 1 respectively. The full operation is shown in Table S3

Table S3. Correlation between Received Classical Value and its Corresponding Unitary Transformations of u_j

Transmitted Bit	$\boldsymbol{u_j}$ *Transformation*
0	I_{u_i}
1	X_{u_i}

Table S2. Correlation between Received Classical Value and its Corresponding Unitary Transformations of u_i

First Bit	*Second Bit*	$\boldsymbol{u_i}$ *Transformation*
0	0	I_{u_i}
0	1	X_{u_i}
1	0	Y_{u_i}
1	1	Z_{u_i}

4. Afterward, the GHZ states will be converted according to transmitted bits, u_i and u_j transformations as shown by equations from Eq. (11−18).

- When the transmitted bits = 000, both u_i and u_j apply I operation on their bits

$$(I_{u_i} \otimes I_{u_j})|\Psi> = \frac{1}{\sqrt{2}} \left(|0000>_{ijql} + |1111>_{ijql}\right)$$

$$= ½ \left\{\left(|\Psi^+>_{ijl} + |\Psi^->_{ijl}\right)|0>_q + \left(|\Psi^+>_{ijl} - |\Psi^->_{ijl}\right)|1>_q\right\}$$

$$= \frac{1}{\sqrt{2}} \left(|\Psi^+>_{ijl} |+>_q + |\Psi^->_{ijl} |->_q\right) \quad (11)$$

- When the transmitted bits = 001, u_i apply I and u_j apply X operations respectively on their bits

$$(I_{u_i} \otimes X_{u_j})|\Psi> = \frac{1}{\sqrt{2}} \left(|0100>_{ijql} + |1011>_{ijql}\right)$$

$$= ½ \left\{\left(|\phi^+>_{ijl} + |\phi^->_{ijl}\right)|0>_q + \left(|\phi^+>_{ijl} - |\phi^->_{ijl}\right)|1>_q\right\}$$

$$= \frac{1}{\sqrt{2}} \left(|\phi^+>_{ijl} |+>_q + |\phi^->_{ijl} |->_q\right) \quad (12)$$

- When the transmitted bits = 010, u_i apply X and u_j apply I operations respectively on their bits

$$(X_{u_i} \otimes I_{u_j})|\Psi> = \frac{1}{\sqrt{2}} \left(|1000>_{ijql} + |0111>_{ijql}\right)$$

$$= ½ \left\{\left(|\psi^+>_{ijl} - |\psi^->_{ijl}\right)|0>_q + \left(|\psi^+>_{ijl} + |\psi^->_{ijl}\right)|1>_q\right\}$$

$$= \frac{1}{\sqrt{2}} \left(|\psi^+>_{ijl} |+>_q - |\psi^->_{ijl} |->_q\right) \quad (13)$$

- When the transmitted bits = 011, both u_i and u_j apply X operation on their bits

$$(X_{u_i} \otimes X_{u_j})|\Psi> = \frac{1}{\sqrt{2}} \left(|1100>_{ijql} + |0011>_{ijql}\right)$$

$$= ½ \left\{\left(|\varphi^+>_{ijl} - |\varphi^->_{ijl}\right)|0>_q + \left(|\varphi^+>_{ijl} + |\varphi^->_{ijl}\right)|1>_q\right\}$$

$$= \frac{1}{\sqrt{2}} \left(|\varphi^+>_{ijl} |+>_q - |\varphi^->_{ijl} |->_q\right) \quad (14)$$

- When the transmitted bits = 100, u_i applies Y and u_j applies I operations respectively on their bits

$$(Y_{u_i} \otimes I_{u_j})|\Psi> = \frac{1}{\sqrt{2}} \left(|0111>_{ijql} - |1000>_{ijql}\right)$$

$$= ½ \left\{\left(|\psi^->_{ijl} - |\psi^+>_{ijl}\right)|0>_q + \left(|\psi^+>_{ijl} + |\psi^->_{ijl}\right)|1>_q\right\}$$

$$= \frac{1}{\sqrt{2}} \left(|\psi^->_{ijl} |+>_q - |\psi^+>_{ijl} |->_q\right) \quad (15)$$

- When the transmitted bits = 101, u_i applies Y and u_j applies X operations respectively on their bits

$$(Y_{u_i} \otimes X_{u_j})|\Psi> = \frac{1}{\sqrt{2}} \left(|0011>_{ijql} - |1100>_{ijql}\right)$$

$$= ½ \left\{\left(|\varphi^->_{ijl} - |\varphi^+>_{ijl}\right)|0>_q + \left(|\varphi^+>_{ijl} + |\varphi^->_{ijl}\right)|1>_q\right\}$$

$$= \frac{1}{\sqrt{2}} \left(|\varphi^->_{ijl} |+>_q - |\varphi^+>_{ijl} |->_q\right) \quad (16)$$

- When the transmitted bits = 110, u_i applies Z and u_j applies I operations respectively on their bits

$$(Z_{u_i} \otimes I_{u_j})|\Psi> = \frac{1}{\sqrt{2}} \left(|0000>_{ijql} - |1111>_{ijql}\right)$$

$$= ½ \left\{\left(|\Psi^+>_{ijl} + |\Psi^+>_{ijl}\right)|0>_q + \left(|\Psi^->_{ijl} + |\Psi^+>_{ijl}\right)|1>_q\right\}$$

$$= \frac{1}{\sqrt{2}} \left(|\Psi^- >_{ijl} |+>_q + |\Psi^+ >_{ijl} |->_q\right) \quad (17)$$

- When the transmitted bits = 111, u_i applies Z and u_j applies X operations respectively on their bits

$$(Z_{u_i} \otimes X_{u_j})|\Psi> = \frac{1}{\sqrt{2}} \left(|0100 >_{ijql} - |1011 >_{ijql}\right)$$

$$= ½ \left\{\left(|\phi^+ >_{ijl} + |\phi^- >_{ijl}\right)|0 >_q + \left(|\phi^- >_{ijl} - |\phi^+ >_{ijl}\right)|1 >_q\right\}$$

$$= \frac{1}{\sqrt{2}} \left(|\phi^- >_{ijl} |+>_q + |\phi^+ >_{ijl} |->_q\right) \quad (18)$$

5. Afterward, both u_i and u_j transmit the transformed message to the received disjoint user u_l, u_l performs a GHZ measurement on his particle , u_i and u_j particles.
6. The quantum server calculates the status of his particle according to x basis $\{+, -\}$ and announces his measurement results.
7. u_l uses his measurements' and the quantum server's publication for retrieving the original sent secret bits by both u_i and u_j.

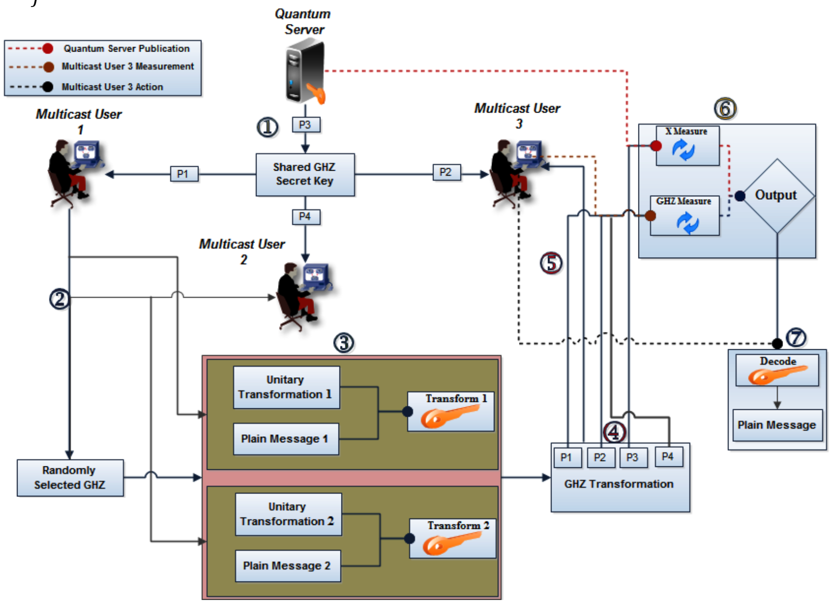

Figure S3: Communication Process between Three Disjoint Users with Partial Support of Quantum Server "drawn by A.F"

2.2 Full Cooperation Process

This process consists of the steps required when two disjointed disjoint users u_i and u_j would like to transmit a secret message to a remote user u_l with full support of the quantum server. In other words the quantum server is functioning as message passing center between the communicated disjoint users. To achieve this function, both u_i and u_j transmit the transformed message to the quantum server instead of u_l . Afterwards, u_l retrieves the original sent secret message according to his publication and quantum server's GHZ measurement on the received transformed message as illustrated in Table S4. Fig. S4 illustrates the flow processes to communicate disjoint user 1 and user 2 with full support of the quantum server. The required steps listed below as the first three steps are same partial mechanism but the process is just changed from step four as indicated in Fig. S4 green box.

4. Afterward, both u_i and u_j transmit the transformed message to the quantum server, quantum server performs a GHZ measurement on his particle, u_i and u_j particles.
5. u_l calculates the status of his particle according to x basis $\{+\,,-\}$ and announces his measurement results.
6. u_l uses his publication and the quantum server's measurement for retrieving the original sent secret bits which sent by both u_i and u_j as shown in Table S4. For example when quantum server measurement is equivalent to $|\phi^{+}>$ and u_l Publication is $|+>$, so u_l can conclude that u_i and u_j applied I and X operations respectively and the sent bits are 001.

Table S4. Correlation between Quantum Server's Measurement, u_l Publication, u_i , u_j Operations and Sent Bits

Quantum Server's Measurement	$\boldsymbol{u_l}$ Publication	$\boldsymbol{u_i}$ Operation	Sent Bits	$\boldsymbol{u_j}$ Operation	Sent Bits	Message Sent
$|\boldsymbol{\Psi}^{+}>_{\boldsymbol{ijq}}$	$|+>_l$	I	00	I	0	000
$|\boldsymbol{\Psi}^{+}>_{\boldsymbol{ijq}}$	$|->_l$	Z	11	I	0	110
$|\boldsymbol{\psi}^{+}>_{\boldsymbol{ijq}}$	$|+>_l$	X	01	I	0	010
$|\boldsymbol{\psi}^{+}>_{\boldsymbol{ijq}}$	$|->_l$	Y	10	I	0	100
$|\boldsymbol{\phi}^{+}>_{\boldsymbol{ijq}}$	$|+>_l$	I	00	X	1	001
$|\boldsymbol{\phi}^{+}>_{\boldsymbol{ijq}}$	$|->_l$	Z	11	X	1	111
$|\boldsymbol{\varphi}^{+}>_{\boldsymbol{ijq}}$	$|+>_l$	X	01	X	1	011
$|\boldsymbol{\varphi}^{+}>_{\boldsymbol{ijq}}$	$|->_l$	Y	10	X	1	101
$|\boldsymbol{\Psi}^{-}>_{\boldsymbol{ijq}}$	$|+>_l$	Z	11	I	0	110
$|\boldsymbol{\Psi}^{-}>_{\boldsymbol{ijq}}$	$|->_l$	I	00	I	0	000
$|\boldsymbol{\psi}^{-}>_{\boldsymbol{ijq}}$	$|+>_l$	Y	10	I	0	100
$|\boldsymbol{\psi}^{-}>_{\boldsymbol{ijq}}$	$|->_l$	X	01	I	0	010
$|\boldsymbol{\phi}^{-}>_{\boldsymbol{ijq}}$	$|+>_l$	Z	11	X	1	111
$|\boldsymbol{\phi}^{-}>_{\boldsymbol{ijq}}$	$|->_l$	I	00	X	1	001
$|\boldsymbol{\varphi}^{-}>_{\boldsymbol{ijq}}$	$|+>_l$	Y	10	X	1	101
$|\boldsymbol{\varphi}^{-}>_{\boldsymbol{ijq}}$	$|->_l$	X	01	X	1	011

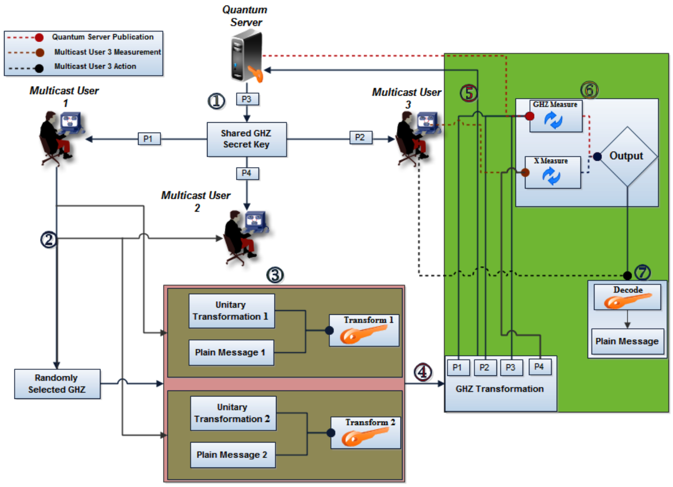

Figure S4: Communication Process between Three Disjoint Users with Full Support of Quantum Server: Green Box indicates Different Steps from Fig. S3 "drawn by A.F"

3 Full Process for Two- Way Channel Substitution Fraudulent Attack

The operation of the attacker Θ_1 and ε on the transmitted particle u is given by (Eq. (19) − (22))

$$|0_u\mathcal{E}> \rightarrow \ \alpha_{\mathcal{E}}|0_u\mathcal{E}_{00}> \ +\beta_{\mathcal{E}}|1_u\mathcal{E}_{01}> \tag{19}$$

$$|1_u\mathcal{E}> \rightarrow \ \beta_{\mathcal{E}}|0_u\mathcal{E}_{10}> \ +\alpha_{\mathcal{E}}|1_u\mathcal{E}_{11}> \tag{20}$$

$$\begin{aligned}|+_u\mathcal{E}> \rightarrow \ & \frac{1}{2}|+_u>(\alpha_{\mathcal{E}}|\mathcal{E}_{00}> + \alpha_{\mathcal{E}}|\mathcal{E}_{11}> + \beta_{\mathcal{E}}|\mathcal{E}_{01}> \ + \beta_{\mathcal{E}}|\mathcal{E}_{10}>) \\ & +\frac{1}{2}|-_u>(\alpha_{\mathcal{E}}|\mathcal{E}_{00}> - \alpha_{\mathcal{E}}|\mathcal{E}_{11}> - \beta_{\mathcal{E}}|\mathcal{E}_{01}> \ + \beta_{\mathcal{E}}|\mathcal{E}_{10}>)\end{aligned} \tag{21}$$

$$\begin{aligned}|-_u\mathcal{E}> \rightarrow \ & \frac{1}{2}|+_u>(\alpha_{\mathcal{E}}|\mathcal{E}_{00}> - \alpha_{\mathcal{E}}|\mathcal{E}_{11}> + \beta_{\mathcal{E}}|\mathcal{E}_{01}> \ - \beta_{\mathcal{E}}|\mathcal{E}_{10}>) \\ & +\frac{1}{2}|-_u>(\alpha_{\mathcal{E}}|\mathcal{E}_{00}> + \alpha_{\mathcal{E}}|\mathcal{E}_{11}> - \beta_{\mathcal{E}}|\mathcal{E}_{01}> \ - \beta_{\mathcal{E}}|\mathcal{E}_{10}>)\end{aligned} \tag{22}$$

Correspondingly, the operation of the attacker Θ_2 and η on the transmitted information particle n is given by (Eq. (23) − (26))

$$|0_n\eta> \rightarrow \ \alpha_\eta|0_n\eta_{00}> \ +\beta_\eta|1_n\eta_{01}> \tag{23}$$

$$|1_n\eta> \rightarrow \ \beta_\eta|0_n\eta_{10}> \ +\alpha_\eta|1_n\eta_{11}> \tag{24}$$

$$\begin{aligned}|+_n\eta> \rightarrow \ & \frac{1}{2}|+_n>(\alpha_\eta|\eta_{00}> + \alpha_\eta|\eta_{11}> + \beta_\eta|\eta_{01}> \ + \beta_\eta|\eta_{10}>) \\ & +\frac{1}{2}|-_n>(\alpha_\eta|\eta_{00}> - \alpha_\eta|\eta_{11}> - \beta_\eta|\eta_{01}> \ + \beta_\eta|\eta_{10}>)\end{aligned} \tag{25}$$

$$\begin{aligned}|-_n\eta> \rightarrow \ & \frac{1}{2}|+_n>(\alpha_\eta|\eta_{00}> - \alpha_\eta|\eta_{11}> + \beta_\eta|\eta_{01}> \ - \beta_\eta|\eta_{10}>) \\ & +\frac{1}{2}|-_n>(\alpha_\eta|\eta_{00}> + \alpha_\eta|\eta_{11}> - \beta_\eta|\eta_{01}> \ - \beta_\eta|\eta_{10}>)\end{aligned} \tag{26}$$

Correspondingly, applying the unitary operation requires the following conditions see (Eq. (27−29))

$$|\alpha_{\mathcal{E}}^2|+|\beta_{\mathcal{E}}^2| = 1,\ |\alpha_\eta^2|+|\beta_\eta^2| = 1 \tag{27}$$

$$<\mathcal{E}_{00}\,|\,\mathcal{E}_{10}>+<\mathcal{E}_{01}\,|\,\mathcal{E}_{11}> = 0 \tag{28}$$

$$<\eta_{00}\,|\,\eta_{10}> + <\eta_{01}\,|\,\eta_{11}> = 0 \tag{29}$$

As well for shorten the discussion, supposes that equations for orthogonal conditions and equations for non-orthogonal conditions are given by (Eq. (30, 31)) and (Eq. (32, 33)) respectively.

$$<\mathcal{E}_{00}\,|\,\mathcal{E}_{01}> = <\mathcal{E}_{10}\,|\,\mathcal{E}_{11}> = <\mathcal{E}_{00}\,|\,\mathcal{E}_{10}> = <\mathcal{E}_{01}\,|\,\mathcal{E}_{11}> = 0 \tag{30}$$

$$<\eta_{00}\,|\,\eta_{01}> = <\eta_{10}\,|\,\eta_{11}> = <\eta_{00}\,|\,\eta_{10}> = <\eta_{01}\,|\,\eta_{11}> = 0 \tag{31}$$

$$<\mathcal{E}_{00}\,|\,\mathcal{E}_{11}> = \cos\theta_{\mathcal{E}}\ ,\ <\mathcal{E}_{01}\,|\,\mathcal{E}_{10}> = \cos\varphi_{\mathcal{E}} \tag{32}$$

$$<\eta_{00}\,|\,\eta_{11}> = \cos\theta_\eta\ ,\ <\eta_{01}\,|\,\eta_{10}> = \cos\varphi_\eta \tag{33}$$

When the two-bit key $A_i \ \ A_{i+1}$ = 00, so the resulting decoding state by the quantum server is given by (Eq. (34, 35))

$$|\Phi_{qu}^{00}> = \ \not{C}_0\,\Theta_2\,\{\,\not{C}_0\,[\Theta_1\,(|\Phi_{qu}^{+}>|\,\mathcal{E}>)\,|\,|\phi_n>]\,|\,\eta>\} \tag{34}$$

$$\begin{aligned}|\Phi_{qu}^{00}> = \ & \frac{1}{\sqrt{2}}(\alpha_{\mathcal{E}}\,\alpha_\eta\,|0_q0_u0_n\,\mathcal{E}_{00}\,\eta_{00}> \ +\alpha_{\mathcal{E}}\,\beta_\eta\,|0_q0_u1_n\,\mathcal{E}_{00}\,\eta_{01}> \\ & +\beta_{\mathcal{E}}\,\beta_\eta|0_q1_u0_n\,\mathcal{E}_{01}\,\eta_{10}> + \beta_{\mathcal{E}}\,\alpha_\eta|0_q1_u1_n\,\mathcal{E}_{01}\,\eta_{11}> \\ & +\beta_{\mathcal{E}}\,\alpha_\eta\,|1_q0_u1_n\,\mathcal{E}_{10}\,\eta_{00}> \ +\beta_{\mathcal{E}}\,\beta_\eta|1_q0_u0_n\,\mathcal{E}_{01}\,\eta_{01}> \\ & +\alpha_{\mathcal{E}}\,\alpha_\eta\,|1_q1_u1_n\,\mathcal{E}_{11}\,\eta_{10}\ \ > + \alpha_{\mathcal{E}}\,\alpha_\eta\,|1_q1_u0_n\ \ \mathcal{E}_{11}\,\eta_{11}>)\end{aligned} \tag{35}$$

The attacker will be discovered if the transmitted particle state is not $|0_n>$, in such situation, the possibility of distinguishing the attacker when $A_i \; A_{i+1} \; = 00$ is given by (Eq. (36))

$$\mathrm{p}_{Total}(A_i \; A_{i+1} \; = 00 \;) = (\alpha_\varepsilon \, \beta_\eta)^2 + (\beta_\varepsilon \, \alpha_\eta)^2 \tag{36}$$

By applying the similar sequences from (Eq. (34) to (36)) for $A_i \; A_{i+1} \; = 01$ showing that the possibility of distinguishing the attacker is equivalent to $\mathrm{p}_{Total}(A_i \; A_{i+1} \; = 00 \;)$. The possibility of distinguishing the attacker when $A_i \; A_{i+1} \; = 10$ is given by (Eq. (37))

$$\mathrm{p}_{Total}(A_i \; A_{i+1} \; = 10 \;) = \frac{1}{2} \, [\, (\alpha_\varepsilon \, \beta_\eta)^2 (\, 1 + \cos \theta_\varepsilon) + (\beta_\varepsilon \, \beta_\eta)^2 (\, 1 + \cos \varphi_\varepsilon) + \left(\alpha_\varepsilon \, \alpha_\eta\right)^2 (\, 1 - \cos \theta_\varepsilon) + \left(\beta_\varepsilon \, \alpha_\eta\right)^2 (\, 1 - \cos \varphi_\varepsilon)] \tag{37}$$

By applying the similar sequences from (Eq. (34) − (36)) for $A_i \; A_{i+1} \; = 11$ showing that the possibility of distinguishing the attacker is equivalent to $\mathrm{p}_{Total}(A_i \; A_{i+1} \; = 10 \;)$. From above equations we can conclude that when $A_i \; = 0$ the possibility of distinguishing the attacker is equivalent to $(\alpha_\varepsilon \, \beta_\eta)^2 + (\beta_\varepsilon \, \alpha_\eta)^2 (\alpha_\varepsilon \, \beta_\eta)^2 + (\beta_\varepsilon \, \alpha_\eta)^2$ and when $A_i \; = 1$ is equivalent to $\frac{1}{2} \, [\, (\alpha_\varepsilon \, \beta_\eta)^2 (\, 1 + cos \; \theta_\varepsilon) + (\beta_\varepsilon \, \beta_\eta)^2 (\, 1 + cos \; \varphi_\varepsilon) + \left(\alpha_\varepsilon \, \alpha_\eta\right)^2 (\, 1 - cos \; \theta_\varepsilon) + \left(\beta_\varepsilon \, \alpha_\eta\right)^2 (\, 1 - cos \; \varphi_\varepsilon)]$ as illustrated in Fig. S5.

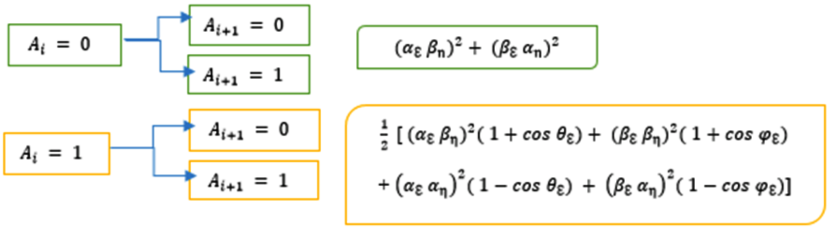

Figure S5: Relation between Two-Bit key $A_i \; A_{i+1}$ and p_{Total}

By combining Eq. (36) and Eq. (37), we can calculate the total possibility for discovering the attacker in the authentication process is given by (Eq. (38))

$$\mathrm{p}_{Total} = ½ \, [\, \mathrm{p}_{Total}(A_i \; = 0 \,) + \mathrm{p}_{Total}(A_i \; = 1 \,)] \tag{38}$$

If the attacker would like to minimize his detection probability then the attacker has to adjust p_{Total} as minimum discovering probability see (Eq. (39)). Eq. (39) calculated under the condition of $\alpha_\varepsilon = \; \alpha_\eta = 1$

$$Total = Min\left(\mathrm{p}_{Total}\right) = \frac{1}{4} \, (1 - cos \; \theta_\varepsilon) \tag{39}$$

From Eq. (39) it's shown that $Min\left(\mathrm{p}_{Total}\right)$ is depends on $cos \; \theta_\varepsilon$ and unrelated to θ_η. So, the attacker's total information amount on the transmitted key bits between the quantum server and the disjoint user can be estimated by (Eq. (40)).

$$\mathfrak{I}(A_K \; , \Theta_{Total} \;) = \sum_{x,y} \mathcal{P}(A_K \; , \Theta_{Total} \;) \log_2 \frac{\mathcal{P}(A_K \; , \Theta_{Total} \;)}{\mathcal{P}(A_K) \, \mathcal{P}(\, \Theta_{Total})} \tag{40}$$

Where Θ_{Total} represents the total operation performed by the attacker Θ_1 and Θ_2 , x represents the key values (00, 01, 10, 11) with probability $\mathcal{P}(x) = \frac{1}{4}$, A_K indicates the selected random values from variable x , $y \; = \mathcal{E}_{ij} \eta_{\mu\tau}$ with $i, j, \mu \, , \tau \; \in \{\, 0 \, , 1\}$ which represents 16 possibilities of the mutual measurement output of the attacker at positions Θ_1 and Θ_2 . For retrieving the value of attacker's total amount of information from Eq. (40). We should only attain the $\mathcal{P}(A_K)$ and $\mathcal{P}(\; \Theta_{Total} \mid A_K \;)$ by (Eq. (41))

$$\mathcal{P}(A_K \; , \Theta_{Total} \;) = \mathcal{P}(\; A_K \;) \, \mathcal{P}(\; \Theta_{Total} \mid A_K \;) \tag{41}$$

Assuming a special case $\mathcal{P}(\; \mathcal{E}_{00} \, \eta_{00} \mid \; 00 \;)$ by substituting in Eq. (35), the minimum detection probability of the attacker's total operation Θ_{Total} is either $\mathcal{E}_{00} \, \eta_{00}$ or $\mathcal{E}_{11} \, \eta_{11}$ with equal possibility of ½ when $A_{2i-1} \, A_{2i} = 00$ see (Eq. (42))

$$|\Phi_{Total}{}^{00}_{qu} > = \frac{1}{\sqrt{2}}(\ |0_q 0_u 0_n\ \mathcal{E}_{00}\,\eta_{00} >\ +\ |1_q 1_u 0_n\ \mathcal{E}_{11}\,\eta_{11} >) \tag{42}$$

Since <$\mathcal{E}_{00}$ | $\mathcal{E}_{11}$> = $cos\ \theta_{\mathcal{E}}$ from Eq. (32) so

$$\text{Ꝑ}(\ \mathcal{E}_{00}\,\eta_{00}\ |\ \ 00\ ) = (1\ +\ sin\ \theta_{\mathcal{E}})\ /\ 2 \tag{43}$$

Since $\text{Ꝑ}(x) = \frac{1}{4}$, so $\text{Ꝑ}(00\ ) = \frac{1}{4}$ and from (Eq. (43)) $\text{Ꝑ}(\ \mathcal{E}_{00}\,\eta_{00}\ |\ \ 00\ )\ =\ (1\ +\ sin\ \theta_{\mathcal{E}})\ /\ 2.$ Therefore by substitution in Eq. (41), the value of attacker's total amount of information on transmitted key values $A_{2i-1}\,A_{2i} = 00$ is given by (Eq. (44))

$$\text{Ꝑ}(\ \ 00\ ,\ \mathcal{E}_{00}\,\eta_{00}\ ) =\ \text{Ꝑ}(00\ )\,\text{Ꝑ}(\ \mathcal{E}_{00}\,\eta_{00}\ |\ \ 00\ ) = \frac{1+sin\ \theta_{\mathcal{E}}}{8} \tag{44}$$

By same way when $A_{2i-1}\,A_{2i} = 01$ the attacker's measurement output can be $\mathcal{E}_{00}\,\eta_{11}$ *or* $\mathcal{E}_{11}\,\eta_{00}$, as well when $A_{2i-1}\,A_{2i} = 10\ |\ 11$ one of four probable outputs $\{\ \mathcal{E}_{00}\,\eta_{00}\ ,\ \mathcal{E}_{00}\,\eta_{11}\ ,\ \mathcal{E}_{11}\,\eta_{00}\ ,\ \mathcal{E}_{11}\,\eta_{11}\ \}$. Consequently, the joint gained information by attacker's total operation Θ_{Total} is given by (Eq. (45))

$$\mathfrak{T} = \frac{1}{4}\ [\ (\ 1 + sin\ \theta_{\mathcal{E}}\ )\ \log_2(\ 1 + sin\ \theta_{\mathcal{E}}\ ) + (\ 1 - sin\ \theta_{\mathcal{E}}\ )\ \log_2(\ 1 - sin\ \theta_{\mathcal{E}}\ )\ ] \tag{45}$$

Since $\sin\theta_{\mathcal{E}} = \sqrt{8 \times Total - 16 \times\ Total^2}$ (see Supplementary information (4) for Proving Relation between $\sin\theta_{\mathcal{E}}$ and $Total$), by substitution in Eq. (45)

$$\mathfrak{T} = \frac{1}{4}\ [\ (\ 1 + \sqrt{8 \times Total - 16 \times\ Total^2})\ \log_2(\ 1 + \sqrt{8 \times Total - 16\ \times Total^2}\ )$$
$$+\ (\ 1 - \sqrt{8 \times Total - 16 \times\ Total^2})\ \log_2(\ 1 - \sqrt{8 \times Total - 16 \times\ Total^2}\ )\ ] \tag{46}$$

In case of the attacker positively receiving the transmission key A_K between the quantum server and disjoint user, so for each transmitted key the attacker has to determine which two-bits are used. As per the attacker's measurement output y = $\mathcal{E}_{ij}\eta_{\mu\tau}$ with $i, j,\ \mu\ , \tau\ \ \in \{\ 0\ ,1\}$ and key values are $(00, 01, 10, 11)$, he can estimate the possibility of the key bits. For instance if y = $\mathcal{E}_{00}\eta_{11}$ then the attacker can estimate that the transmitted key bits either 00, 10 or 11 with occurrence possibility 0.5, 0.25 and 0.25 correspondingly. By assuming that the attacker chooses the possibility for detecting transmitted key values 00 or 01 is Ꝑ and for detecting 10 and 11 is $1 -$Ꝑ and take into account the inconclusive measurement output as in Eq. (43). Therefore the total estimation probability Ꝑ_e of A_K is given by (Eq. (47))

$$\text{Ꝑ}_e = \frac{(1 + sin\ \theta_{\mathcal{E}})}{2}\left[\frac{1}{2}\ \text{Ꝑ} + \frac{1}{4}\ (1 - \text{Ꝑ})\right] + \frac{(1 - sin\ \theta_{\mathcal{E}})}{2}\left[\frac{1}{4}\ (1 - \text{Ꝑ})\right] \tag{47}$$

By simplification of Eq. (47) Ꝑ_e of A_K is given by (Eq. (48)) (see Supplementary information (5) for Proving Relation between Ꝑ_e , Ꝑ and $\mathbf{sin}\ \boldsymbol{\theta}_{\mathcal{E}}$)

$$\text{Ꝑ}_e = \frac{1}{8}[\ (\ sin\ \theta_{\mathcal{E}}(3 \times \text{Ꝑ} - 1\ ) + 2] \tag{48}$$

If $\text{Ꝑ} = 1$ indicates that the total estimation probability Ꝑ_e is maximized see (Eq. (49)) (see Supplementary information (6) for Proving Relation between Ꝑ_e ,$\text{Ꝑ}_e{}^m$ and $Total$)

$$\text{Ꝑ}_e{}^m = \frac{1}{4}\ (\ \sqrt{8 \times Total - 16 \times\ Total^2}\ + 1) \tag{49}$$

Therefore, the probability of the attacker for successfully retrieving the transmitted keys $\text{Ꝑ}_e{}^r\ for\ A_k\ =$ $\{\ A_1, A_2, A_3\ \dots \dots \dots \dots \dots \dots \dots \dots \dots \dots \dots\ \ A_{2N}\}$ see (Eq. (50))

$$\text{Ꝑ}_e{}^r\ = [\text{Ꝑ}_e{}^m(\ 1 - Total)]^{N/2} \tag{50}$$

By substituting (Eq. (49)) in equation (Eq. (50)), so

$$\text{Ꝑ}_e{}^r\ =\ \left[\frac{1}{4}(\ \sqrt{8 \times Total - 16 \times\ Total^2}\ + 1)(\ 1 - Total)\right]^{N/2} \tag{51}$$

4 Proving Relation between sin $\boldsymbol{\theta_{\varepsilon}}$ and $\boldsymbol{Total}$

From Eq. (39)
$$Total = Min(p_{Total}) = \frac{1}{4}\,(1 - cos\ \theta_{\varepsilon}) \tag{52}$$

$$Total = \frac{1}{4} - \frac{1}{4} cos\ \theta_{\varepsilon} \tag{53}$$

$$4 \times Total = 1 - cos\ \theta_{\varepsilon} \tag{54}$$

$$cos\ \theta_{\varepsilon} = 1 - 4 \times Total \tag{55}$$

$$cos\ \theta_{\varepsilon}{}^{2} = [1 - 4 \times Total]^{2} \tag{56}$$

$$cos\ \theta_{\varepsilon}{}^{2} = [1 - 8 \times Total + 16 \times Total^{2}] \tag{57}$$

By using the mathematical formulation of

$$cos\ \theta_{\varepsilon}{}^{2} + sin\ \theta_{\varepsilon}{}^{2} = 1 \tag{58}$$

Therefore, by substituting from Eq. (57) in Eq. (58)

$$1 - 8 \times Total + 16 \times Total^{2} + sin\ \theta_{\varepsilon}{}^{2} = 1 \tag{59}$$

$$sin\ \theta_{\varepsilon}{}^{2} = 1 - 1 + 8 \times Total - 16 \times Total^{2} \tag{60}$$

$$sin\ \theta_{\varepsilon}{}^{2} = 8 \times Total - 16 \times Total^{2} \tag{61}$$

So

$$sin\ \theta_{\varepsilon} = \sqrt{8 \times Total - 16 \times Total^{2}} \tag{62}$$

5 Proving Relation between Ƥ$_{\boldsymbol{e}}$, Ƥ and sin $\boldsymbol{\theta}_{\varepsilon}$

$$Ƥ_{e} = \frac{(1 + sin\ \theta_{\varepsilon})}{2}\left[\frac{1}{2}\ Ƥ + \frac{1}{4}\ (1 - Ƥ)\right] + \frac{(1 - sin\ \theta_{\varepsilon})}{2}\left[\frac{1}{4}\ (1 - Ƥ)\right] \tag{63}$$

By dividing Eq. (63) into Eq. (64) and Eq. (65)

$$\frac{(1 + sin\ \theta_{\varepsilon})}{2}\left[\frac{1}{2}\ Ƥ + \frac{1}{4}\ (1 - Ƥ)\right] \tag{64}$$

$$\frac{(1 - sin\ \theta_{\varepsilon})}{2}\left[\frac{1}{4}\ (1 - Ƥ)\right] \tag{65}$$

By simplify Eq. (64)

$$\frac{(Ƥ + Ƥ\ sin\ \theta_{\varepsilon})}{4} + \frac{(1 + sin\ \theta_{\varepsilon})(1 - Ƥ)}{8} \tag{66}$$

$$\frac{(Ƥ + Ƥ\ sin\ \theta_{\varepsilon})}{4} + \frac{(1 - Ƥ + sin\ \theta_{\varepsilon} - Ƥ\ sin\ \theta_{\varepsilon})}{8} \tag{67}$$

$$\frac{(2 \times Ƥ + 2 \times Ƥ\ sin\ \theta_{\varepsilon} + 1 - Ƥ + sin\ \theta_{\varepsilon} - Ƥ\ sin\ \theta_{\varepsilon})}{8} \tag{68}$$

By simplify Eq. (65)

$$\frac{(1 - sin\ \theta_{\varepsilon})(1 - Ƥ)}{8} \tag{69}$$

$$\frac{(1 - Ƥ - sin\ \theta_{\varepsilon} + Ƥ\ sin\ \theta_{\varepsilon})}{8} \tag{70}$$

By adding Eq. (68) and Eq. (70)

$$'P_e = \frac{[2+ \sin\theta_{\varepsilon}(2\times 'P+'P-1)]}{8} \qquad (71)$$

$$'P_e = \frac{[2+ \sin\theta_{\varepsilon}(2\times 'P+'P-1)]}{8} \qquad (72)$$

$$'P_e = \frac{1}{8}[\,(\,\sin\theta_{\varepsilon}(3\times 'P-1\,)+2] \qquad (73)$$

So Eq. (73) = Eq. (48)

6 Proving Relation between $'P_e$, $'P_e{}^m$ and $Total$

From Eq. (73) $\qquad 'P_e = \frac{1}{8}[\,(\,\sin\theta_{\varepsilon}(3\times 'P-1\,)+2]$

If $'P = 1$ indicates that the total estimation probability $'P_e$ is maximized to $'P_e{}^m$

$$'P_e{}^m = \frac{1}{8}[\,(\,\sin\theta_{\varepsilon}((3\times 1)-1\,)+2] \qquad (74)$$

$$'P_e{}^m = \frac{1}{8}[\,(\,2\times \sin\theta_{\varepsilon})+2] \qquad (75)$$

$$'P_e{}^m = \frac{2}{8}[\,\sin\theta_{\varepsilon}+1] \qquad (76)$$

$$'P_e{}^m = \frac{1}{4}[\,\sin\theta_{\varepsilon}+1] \qquad (77)$$

From Eq. (39) Appendix 2, $\sin\theta_{\varepsilon} = \sqrt{8\times Total - 16\times\ Total^2}$, by substitution in Eq. (77)

$$'P_e{}^m = \frac{1}{4}\left[\,\sqrt{8\times Total - 16\times\ Total^2}+1\right] \qquad (76)$$

So Eq. (76) = Eq. (49)

7 Full Calculations for Relation between N, $Total$, $'P_e{}^r$

A

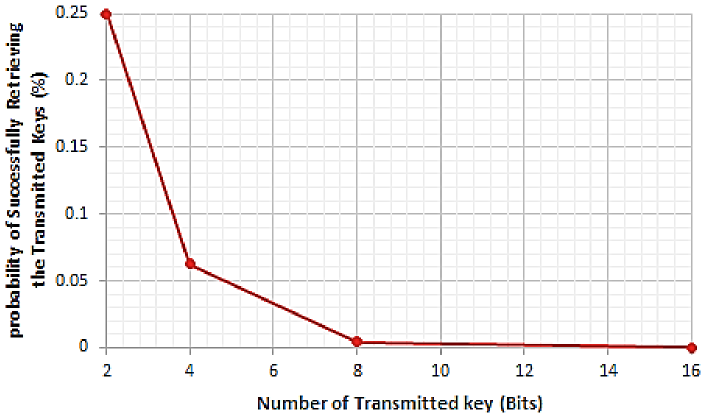

B

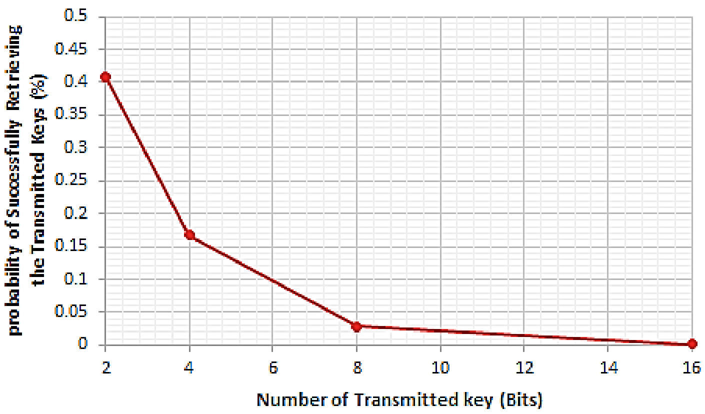

C

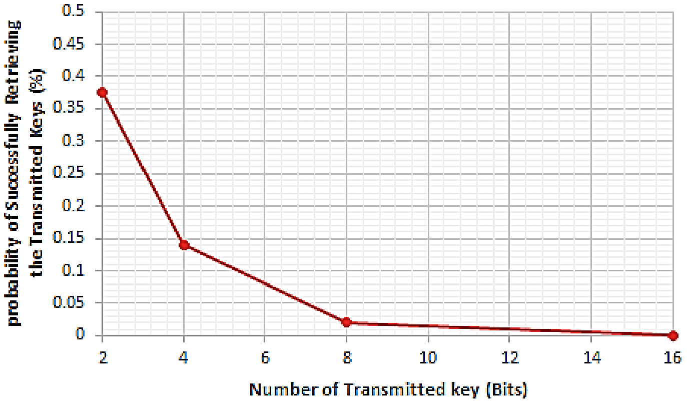

D

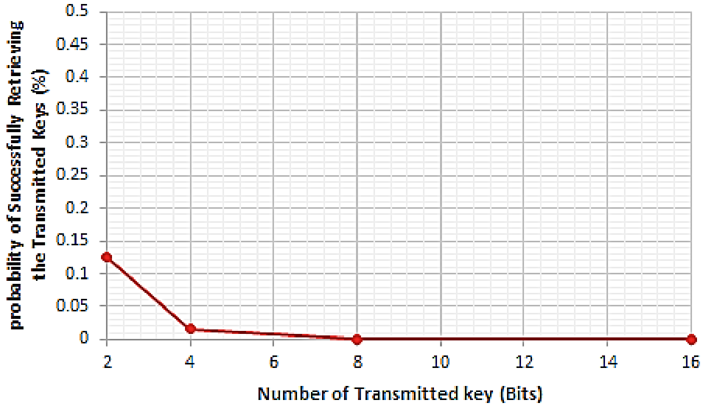

Figure S6: (A) Relation between $Ƥ_e{}^r$, $N =$ [2 ,4 , 8 , 16] and $Total = [\ 0\ ]\%$; **(B)** $Total = [\ 12.5\ ]\%$; **(C)** $Total = [\ 25\ ]\%$; **(D)** $Total = [\ 50\ ]\%$ “drawn by A.F”

Table S5. (A, B, C, D) Numerical Calculations for Fig. S6 ((A), (B), (C), (D))

A

$Total$ (%)	N (Bits)	$Ƥ_e{}^r$
0	2	0.25
0	4	6.25×10^{-2}
0	8	3.91×10^{-3}
0	16	1.53×10^{-5}

B

$Total$ (%)	N (Bits)	$Ƥ_e{}^r$
12.5	2	4.08×10^{-1}
12.5	4	1.668×10^{-1}
12.5	8	2.782×10^{-2}
12.5	16	7.7×10^{-4}

C

$Total$ (%)	N (Bits)	$Ƥ_e{}^r$
25	2	0.375
25	4	1.406×10^{-1}
25	8	1.97×10^{-2}
25	16	3.91×10^{-4}

D

$Total$ (%)	N (Bits)	$Ƥ_e{}^r$
50	2	1.25×10^{-1}
50	4	1.56×10^{-2}
50	8	2.5×10^{-4}
50	16	5.96×10^{-8}

8 Full Calculations for Relation between $Ƥ_e{}^r$ and N while $Ƥ_e{}^m = [12.5\ , 25, 37.5\ , 50\ ]\%$ and $Total = [0, 12.5, 25, 50\ ]\%$

A

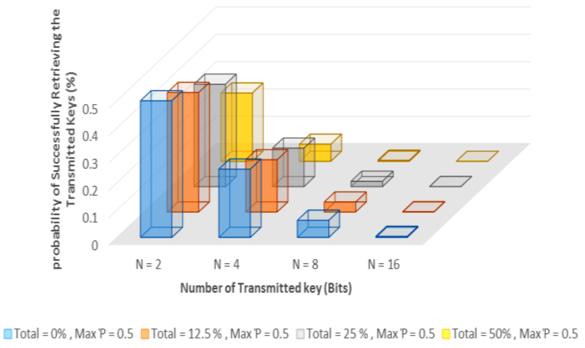

B

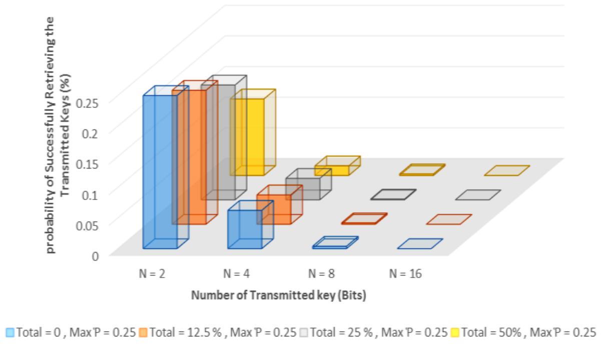

C

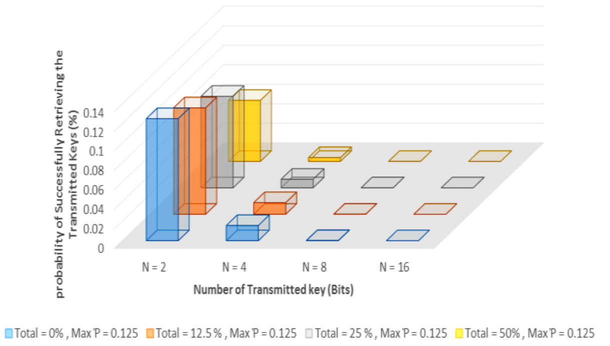

D

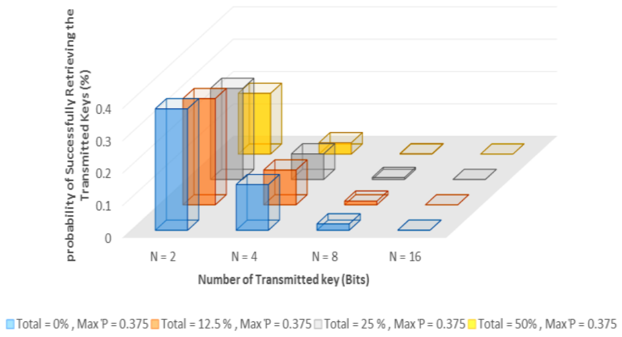

Figure S7: (A) Relation between ‘$P_e{}^r$, $N =$ [2 ,4 , 8 , 16] while $Total$ = [0,12.5,25,50]% and ‘$P_e{}^m$ = [50]% **(B)** ‘$P_e{}^m$ = [25]% ; **(C)** ‘$P_e{}^m$ = [12.5]% ; **(D)** ‘$P_e{}^m$ = [37.5]% “drawn by A.F”

Table S6. (A, B, C, D) Numerical Calculations for Fig. S7 ((B), (A), (C), (D)) respectively

A

‘$P_e{}^m$	$Total$ (%)	N (Bits)	‘$P_e{}^r$
0.25	0	2	0.25
0.25	0	4	0.0625
0.25	0	8	3.91×10^{-3}
0.25	0	16	1.53×10^{-5}
0.25	12.5	2	0.21875
0.25	12.5	4	0.04785
0.25	12.5	8	2.28×10^{-3}
0.25	12.5	16	5.24×10^{-6}
0.25	25	2	0.1875
0.25	25	4	0.0351
0.25	25	8	1.23×10^{-3}
0.25	25	16	1.53×10^{-6}
0.25	50	2	0.125
0.25	50	4	0.015625
0.25	50	8	2.44×10^{-4}
0.25	50	16	5.96×10^{-8}

B

‘$P_e{}^m$	$Total$ (%)	N (Bits	‘$P_e{}^r$
0.50	0	2	0.5
0.50	0	4	0.25
0.50	0	8	0.0625
0.50	0	16	3.91×10^{-3}
0.50	12.5	2	0.4375
0.50	12.5	4	0.1914
0.50	12.5	8	0.0366
0.50	12.5	16	1.34×10^{-3}
0.50	25	2	0.375
0.50	25	4	0.1406
0.50	25	8	0.0197
0.50	25	16	3.91×10^{-4}
0.50	50	2	0.25
0.50	50	4	0.0625
0.50	50	8	3.91×10^{-3}
0.50	50	16	1.53×10^{-5}

C

‘$P_e{}^m$	$Total$ (%)	N (Bits)	‘$P_e{}^r$
0.125	0	2	0.125
0.125	0	4	0.0156
0.125	0	8	2.5×10^{-4}
0.125	0	16	5.96×10^{-8}
0.125	12.5	2	0.109
0.125	12.5	4	0.0119
0.125	12.5	8	1.43×10^{-4}
0.125	12.5	16	2.04×10^{-8}
0.125	25	2	0.09375
0.125	25	4	8.78×10^{-3}
0.125	25	8	7.72×10^{-5}
0.125	25	16	5.96×10^{-9}

D

‘$P_e{}^m$	$Total$ (%)	N (Bits	‘$P_e{}^r$
0.375	0	2	0.375
0.375	0	4	0.1406
0.375	0	8	0.0197
0.375	0	16	3.91×10^{-4}
0.375	12.5	2	0.3281
0.375	12.5	4	0.10766
0.375	12.5	8	0.01159
0.375	12.5	16	1.34×10^{-3}
0.375	25	2	0.28125
0.375	25	4	0.07910
0.375	25	8	6.25×10^{-3}
0.375	25	16	3.91×10^{-5}

0.125	50	2	0.0625
0.125	50	4	3.91×10^{-3}
0.125	50	8	1.53×10^{-5}
0.125	50	16	2.32×10^{-10}

0.375	50	2	0.1875
0.375	50	4	0.03515
0.375	50	8	1.32×10^{-3}
0.375	50	16	1.53×10^{-6}

9 Full Calculations for Relation between $Ƥ_e^{\,r}$ and N while $Ƥ_e^{\,m} = [12.5\,, 25, 37.5\,, 50\,]\%$ and $Total = [62.5,75,87.5\,]\%$

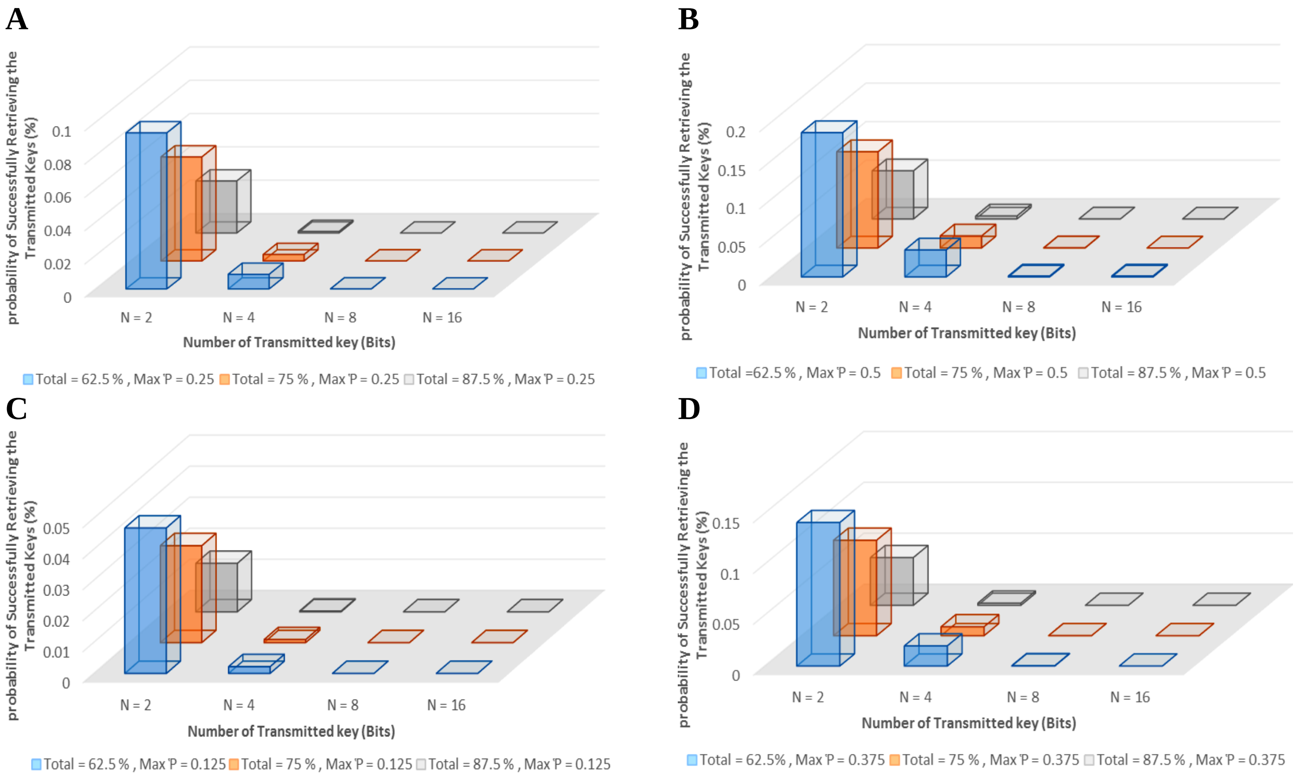

Figure S8: (A) Relation between $Ƥ_e^{\,r}$, $N = [2\,,4\,,8\,,16]$ while $Total = [62.5,75,87.5\,]\%$ and $Ƥ_e^{\,m} = [\,25\,]\%$ **(B)** $Ƥ_e^{\,m} = [\,50\,]\%$; **(C)** $Ƥ_e^{\,m} = [\,12.5\,]\%$; **(D)** $Ƥ_e^{\,m} = [\,37.5\,]\%$ "drawn by A.F"

Table S7. (A, B, C, D) Numerical Calculations for Fig. S8 ((C), (A), (D), (B)) respectively

A

$Ƥ_e^{\,m}$	$Total$ (%)	N (Bits)	$Ƥ_e^{\,r}$
0.125	62.5	2	4.68×10^{-2}
0.125	62.5	4	2.19×10^{-3}
0.125	62.5	8	4.82×10^{-6}
0.125	62.5	16	2.33×10^{-11}
0.125	75	2	3.125×10^{-2}
0.125	75	4	9.77×10^{-4}
0.125	75	8	9.54×10^{-7}
0.125	75	16	9.095×10^{-13}
0.125	87.5	2	0.015625
0.125	87.5	4	2.5×10^{-4}
0.125	87.5	8	5.96×10^{-8}
0.125	87.5	16	3.55×10^{-15}

B

$Ƥ_e^{\,m}$	$Total$ (%)	N (Bits)	$Ƥ_e^{\,r}$
0.25	62.5	2	9.37×10^{-2}
0.25	62.5	4	8.79×10^{-3}
0.25	62.5	8	7.7×10^{-5}
0.25	62.5	16	5.96×10^{-9}
0.25	75	2	0.0625
0.25	75	4	3.91×10^{-3}
0.25	75	8	1.53×10^{-5}
0.25	75	16	2.33×10^{-10}
0.25	87.5	2	0.03125
0.25	87.5	4	9.77×10^{-4}
0.25	87.5	8	9.54×10^{-7}
0.25	87.5	16	9.095×10^{-13}

C

P_e^m	$Total$ (%)	N ($Bits$)	P_e^r
0.375	62.5	2	1.41×10^{-1}
0.375	62.5	4	1.97×10^{-2}
0.375	62.5	8	3.91×10^{-4}
0.375	62.5	16	1.53×10^{-7}
0.375	75	2	9.37×10^{-2}
0.375	75	4	8.79×10^{-3}
0.375	75	8	7.7×10^{-5}
0.375	75	16	5.96×10^{-9}
0.375	87.5	2	4.68×10^{-2}
0.375	87.5	4	2.19×10^{-3}
0.375	87.5	8	4.82×10^{-6}
0.375	87.5	16	2.33×10^{-11}

D

P_e^m	$Total$ (%)	N ($Bits$)	P_e^r
0.50	62.5	2	1.87×10^{-1}
0.50	62.5	4	3.51×10^{-2}
0.50	62.5	8	1.23×10^{-3}
0.50	62.5	16	1.53×10^{-6}
0.50	75	2	0.125
0.50	75	4	0.015625
0.50	75	8	2.5×10^{-4}
0.50	75	16	5.96×10^{-8}
0.50	87.5	2	0.0625
0.50	87.5	4	3.91×10^{-3}
0.50	87.5	8	1.53×10^{-5}
0.50	87.5	16	2.33×10^{-10}
